# Strain-induced topological phase transition with inversion of the in-plane electric polarization in tiny-gap semiconductor SiGe monolayer

**DOI:** 10.1038/s41598-020-68228-3

**Published:** 2020-07-09

**Authors:** Kyu Won Lee, Cheol Eui Lee

**Affiliations:** Department of Physics, Korea University, Seoul, 02841 Republic of Korea

**Keywords:** Materials science, Physics

## Abstract

Our density functional theory calculations show that tiny-gap semiconductor SiGe monolayer is a quantum valley Hall insulator with a spontaneous electric polarization and, under a small biaxial strain, undergoes a topological phase transition between the states with opposite valley Chern numbers. The topological phase transition entails abrupt inversion of the in-plane electric polarization corresponding to inversion of the sublattice pseudospin polarization, while the out-of-plane electric polarization shows a linear response to the biaxial strain as well as to the perpendicular electric field regardless of the phase transition. Thus, the quantum valley Hall state entails in-plane ferroelectricity corresponding to a sublattice pseudospin ferromagnetism.

After the discovery of graphene,^1–3^ two-dimensional (2D) materials have attracted more and more research interest.^4–6^ Graphene, a single layer of carbon atoms arranged in a honeycomb lattice, is a semimetal with linear crossing bands at the K point of the Brillouin zone.^2^ The low-energy physics of graphene can be described by the 2D Dirac equation, and graphene is a quantum spin Hall insulator under spin–orbit coupling.^2, 3^ Monatomic 2D materials of group IV elements such as silicene and germanene are analogous to graphene with a buckled honeycomb lattice. Like graphene, silicene and germanene are semimetals with linear crossing bands and are quantum spin Hall insulators under spin–orbit coupling.^7–9^

Most diatomic 2D materials composed of group IV or group III–V elements have been reported to be wide band gap semiconductors due to their ionic character.^5, 6, 10, 11^ Unlike the other diatomic 2D materials, SiGe monolayer was reported to be a semimetal or a tiny-gap semiconductor with linear energy dispersion near the K point, which was attributed to a very small difference in the electronegativity of Si and Ge elements.^5, 12^ In the presence of spin–orbit coupling, a truly disordered SiGe monolayer was predicted to be a quantum spin Hall insulator from the adiabatic continuity arguments and from a W-shaped behavior of the band gap under an electric field perpendicular to the layer as observed in silicene and germanene.^13^ The ordered SiGe monolayer was reported not to be a quantum spin Hall insulator because the band gap did not show the W-shaped behavior.^13^

Under a small biaxial strain that can be induced by a lattice constant mismatch with the substrate, monatomic 2D group IV materials such as graphene, silicene and germanene show no significant changes in the band structure and the linear energy dispersion near the K point remains almost intact, because the biaxial strain maintains all crystal symmetries.^14, 15^ Uniaxial strain further destroys the symmetry and some conflicting results were reported for the band gap of the uniaxially strained monatomic 2D materials of group IV elements.^16–19^ In a single-orbital tight-binding model, it has been shown that uniaxial strain as well as biaxial strain do not open a band gap in the graphene-like honeycomb lattice.^14^ The band gap of SiGe monolayer was reported to increase with uniaxial strain whether compressive or tensile.^20^ Unlike the monatomic 2D group IV materials, diatomic 2D materials such as SiGe monolayer have broken inversion symmetry and the inversion symmetry-breaking potential may depend on the strain, leading to a change in the band gap.

On the other hand, electron-electron interaction in 2D materials much stronger than that in three-dimensional materials was predicted to lead to an in-plane spontaneous electric polarization.^21^ In monolayer Group-IV monochalcogenides *MX* ($M={\hbox {Ge}}$, Sn; $X={\hbox {S}}$, Se), in-plane displacement of atoms was reported to induce an in-plane spontaneous electric polarization.^22^ In buckled 2D materials, such as ${{\hbox {GaAsC}}}_6$ and diatomic 2D materials of group IV or group III–V elements, out-of-plane displacement of atoms was reported to induce an out-of-plane spontaneous electric polarization whose magnitude and sign depend on the displacement.^23, 24^ In the buckled 2D materials, the two symmetry-equivalent energy minima with opposite polarities are obtained by reversing the buckling angle.

In a (buckled) 2D honeycomb lattice, there exists a sublattice pseudospin in addition to the spin and valley degrees of freedom.^3, 9^ The pseudospin up (down) refers to the state where the charge carriers are located in the A (B) sublattice, or equivalently in the upper (lower) layer because the A and B sublattices are on different layers in the buckled lattice. Sublattice symmetry-breaking leads to a nonzero polarization of the sublattice pseudospin, directly corresponding to an electric polarization. Smooth elastic deformation in graphene and electron-electron interactions in bilayer graphene have been reported to be able to break the sublattice symmetry, leading to pseudospin ferromagnetism corresponding to a ferroelectric state.^25, 26^ It is not clear whether the sublattice pseudospin polarization directly corresponds to the out-of-plane electric polarization, which was attributed to out-of-plane displacement of atoms, in buckled 2D materials.^23, 24^

In a (buckled) 2D honeycomb lattice,^9, 27^ the low-energy dispersion near the Fermi level (${E_F}$) has been well described by a 2D Dirac hamiltonian with a mass gap, ${H=v_{F}(\tau \sigma _{x}k_{x}+\sigma _{y}k_{y})+m\sigma _{z}}$, where ${v_F}$ is the Fermi velocity, $\tau =\pm \,1$ is a valley index, and ${\sigma }$ is the Pauli matrix accounting for the sublattice pseudospin. The mass term ${m\sigma _z}$ can be introduced by an inversion symmetry-breaking potential such as staggered AB-sublattice potentials and electric fields perpendicular to the layer. The broken inversion symmetry in a 2D honeycomb lattice leads to a quantum valley Hall effect, where carriers in different valleys flow to the opposite transverse edges.^9, 27, 28^ The valley Chern number is given by $C_{V}={\hbox {sign}}(m)$, neglecting the spin degeneracy, and the sublattice pseudospin has a nonzero polarization whose sign is also determined by sign(*m*). Thus, the quantum valley Hall state will have spontaneous electric polarization, which directly corresponds to the sublattice pseudospin polarization and is independent of the ion displacement in contrast to the electric polarization reported in some 2D materials.^22–24^

In this work, we have investigated tiny-gap semiconductor SiGe monolayer by using the density functional theory (DFT) calculations. First, we show that the SiGe monolayer is a quantum valley Hall insulator and undergoes a topological phase transition between the quantum valley Hall states with opposite valley Chern numbers under a small biaxial strain or under a perpendicular electric field. Strain-dependent on-site Coulomb potentials of Si-3*p* and Ge-4*p* orbitals seem to be responsible for the quantum valley Hall state and the topological phase transition. Next, we show that the quantum valley Hall state has in-plane ferroelecticity. The topological phase transition entails abrupt inversion of the in-plane electric polarization, while the out-of-plane electric polarization shows a linear response to the biaxial strain as well as to the perpendicular electric field regardless of the phase transition. The in-plane electric polarization is a purely electronic one and directly corresponds to the sublattice pseudospin polarization.

Figure 1a shows the structure of SiGe monolayer. The lattice vectors are ${\vec {a}=a{\hat{x}}}$, ${\vec {b}=a({\hat{x}}/2+\sqrt{3}{\hat{y}}/2)}$ and ${\vec {c}=c{\hat{z}}}$, where *c* was set to 2.6 nm corresponding to the vacuum spacing. The Si and Ge atoms are located at (1/3, 1/3, ${-h}$) and ($2/3, 2/3, {+}h$), respectively, in the fractional coordinates. The equilibrium lattice constant ${a_0} =0.3945 \, {\hbox {nm}}$ was obtained from a total energy minimum as shown in Fig. 1b, in agreement with a previous work.^12^ The buckling height 2*hc*, which gradually decreases with increasing lattice constant, was 0.065 nm at equilibrium, and was insensitive to the perpendicular electric field investigated in this work.

**Figure 1:**
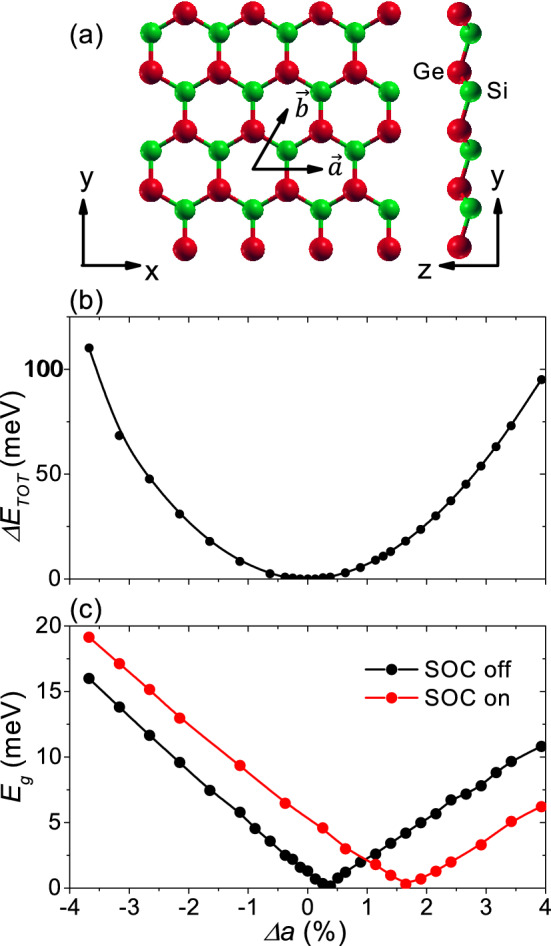
SiGe monolayer. (**a**) Geometric structure. Red and green balls correspond to germanium and silicon, respectively. (**b**) Total energy ${{\Delta }E_{TOT}}$ with respect to the equilibrium value as a function of the biaxial strain ${\Delta a}$. (**c**) Band gap ${E_g}$ as a function of ${\Delta a}$. Black and red symbols correspond to the band gap without and with spin–orbit coupling, respectively.

Figure 1c shows the band gap ${E_g}$ without (black symbols) and with spin–orbit coupling (red symbols) as a function of the biaxial strain ${\Delta a}$. Biaxial in-plane strain ${{\Delta }a}$ was defined by ${{\Delta }a=(a-a_{0})/a_{0}\times 100}$ in units of %, where *a* (${a_0}$) is a strained (equilibrium) lattice constant. We can see that the band gap closes at the critical strain ${\Delta a_c} =0.38\%$ corresponding to ${\textit{a}}=0.396$ nm, indicating a quantum phase transition. The spin–orbit coupling slightly increases the band gap and just shifts the critical strain. In a previous work, the ordered SiGe monolayer corresponding to our case was reported not to be a quantum spin Hall insulator even in the presence of spin–orbit coupling.^13^ The spin–orbit coupling, which is not directly related to the quantum phase transition in Fig. 1c, is neglected in this work.

Figure 2 shows the band structure and Berry curvature (a) for $\Delta a=- \, 1.14\% \, ({<\Delta a_c})$ and (b) for $\Delta a=+\, 1.39\% \, (>\Delta a_c)$, both corresponding to $a =0.39 \, {\hbox {nm}}$ and 0.4 nm. Under small strains investigated in this work, the band structure (black lines) does not largely change and has a small band gap at the K point. At the critical strain, the band structure has linear crossing bands at the K point. The Berry curvature (red lines) shows sharp peaks at the K points with opposite signs at opposite valleys, possibly indicating a valley Hall effect, and the Berry curvature peaks are inverted at the critical strain. The valley Chern number obtained by integrating the Berry curvature was $C_{V}=+\, 1$ for ${{\Delta }a<{\Delta }a_c}$ and $C_{V}=-\, 1$ for ${{\Delta }a>{\Delta }a_c}$, indicating that the quantum phase transition at the critical strain is a topological phase transition between the quantum valley Hall states with opposite valley Chern numbers.

**Figure 2:**
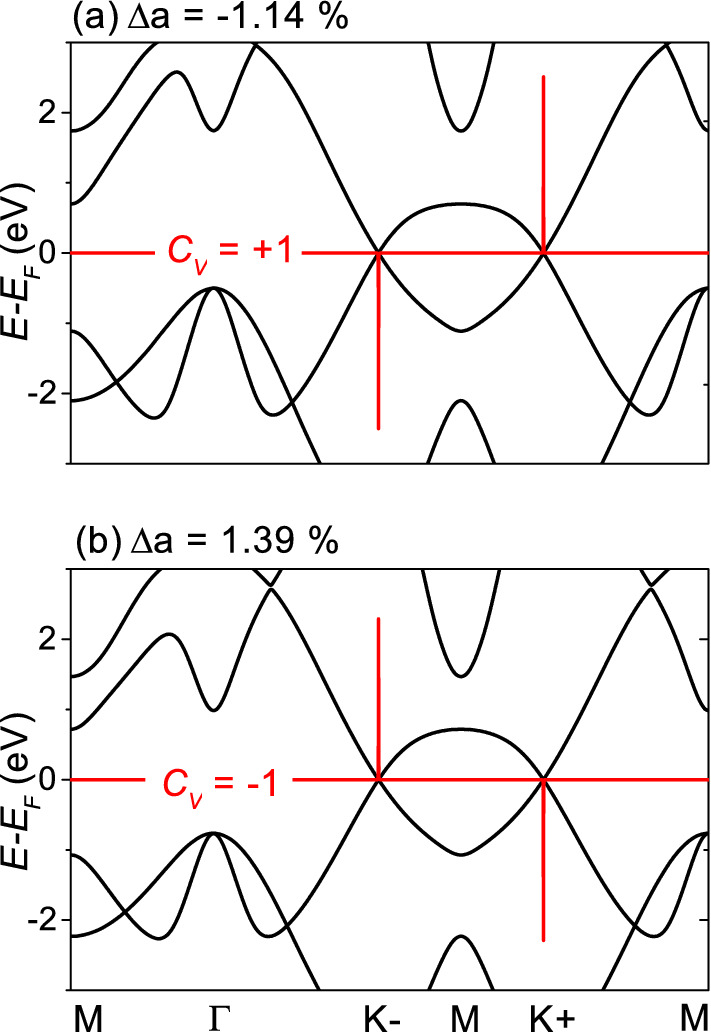
Band structure (black lines) and Berry curvature (red lines). (**a**) $\Delta a=-\, 1.14\% \, ({<\Delta a_c})$. (**b**) $\Delta a= 1.39\% \, ({>\Delta a_c})$. The valley Chern number ${C_{V}}$ was ${+}\, 1$ and ${-}\, 1$ for $\Delta a=-\, 1.14\%$ and 1.39%, respectively.

An external electric field perpendicular to the layer induces a potential difference between the A and B sublattices and can induce the topological phase transition between the quantum valley Hall states. Figure 3a shows the band gap as a function of the perpendicular electric field ${E_z}$ for $\Delta a=- \, 1.14\%$ (solid symbols) and for $\Delta a= 1.39\%$ (open symbols), where we can see the band gap closure corresponding to the topological phase transition. Figure 3b shows the critical electric field ${E_{zc}}$, where the band gap closes, as a function of ${\Delta a}$. ${E_{zc}}$ linearly decreases with increasing lattice constant and is nearly zero at the critical strain, indicating that an intrinsic potential difference between the two sublattices corresponds to the quantum valley Hall states and the strain-induced topological phase transition.

**Figure 3:**
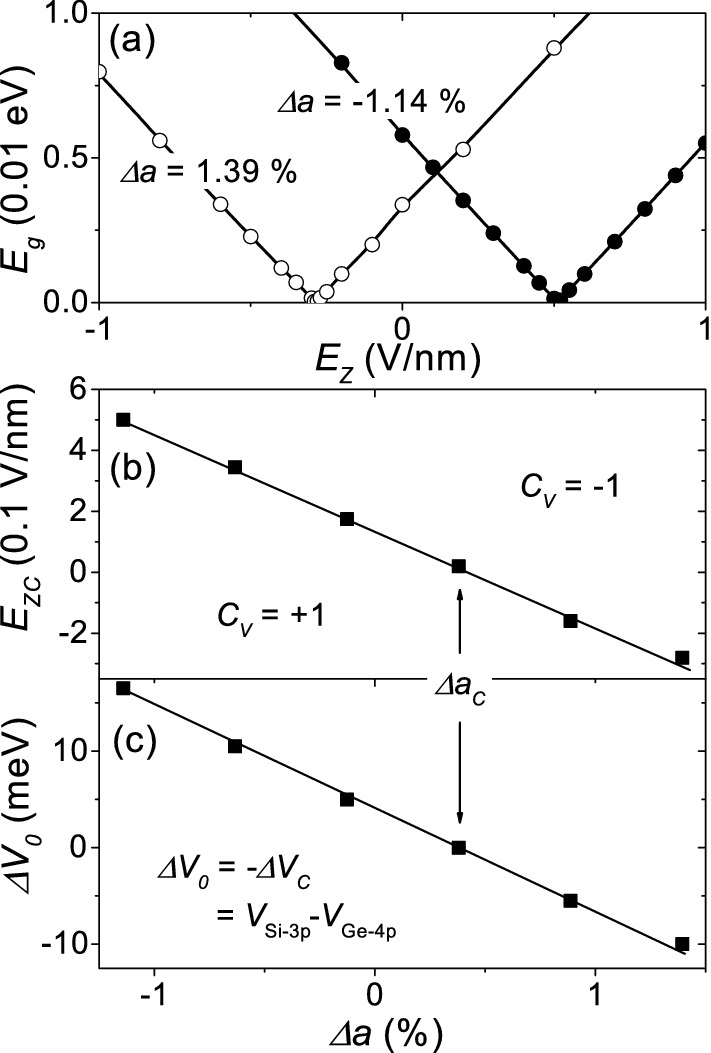
Band gap and intrinsic potential difference. (**a**) Band gap ${E_g}$ as a function of ${E_z}$ for $\Delta a=-\, 1.14\%$ (solid symbols) and for $\Delta a= 1.39\%$ (open symbols). (**b**) Critical electric field ${E_{zc}}$, where the band gap closes, as a function of ${{\Delta }a}$. (**c**) Intrinsic potential difference ${\Delta V_0}$ between Si-3*p* and Ge-4*p* orbitals as a function of ${{\Delta }a}$.

Using a Bader charge analysis, we found that a charge of 0.23*e* is transferred to the silicon atom at equilibrium. The amount of charge transferred to the Si atom monotonically decreases from 0.28*e* at $\Delta a=- \, 1.14\%$ to 0.19*e* at $\Delta a=1.39\%$, and the direction of charge transfer does not change at the topological phase transition. Using a Mulliken population analysis, we obtained essentially the same results, except that the amount of charge transferred to the Si atom is about four times smaller than in the Bader analysis. Although there is some ambiguity in the estimation of the effective charge, the band gap change leading to the topological phase transition seems to be independent of the charge transfer.

To estimate the intrinsic potential difference ${{\Delta }V_{0}}$ between the two sublattices, a potential difference ${{\Delta }V}$ between the Ge-4*p* and Si-3*p* orbitals was applied (see Methods section). ${{\Delta }V}$ was controlled to reduce the band gap down to zero. The critical value ${{\Delta }V_C}$, where the band gap closes, can be considered as the counterbalance of the intrinsic potential difference between the Ge-4*p* and Si-3*p* orbitals, i.e., ${{\Delta }V_{0}=- \,{\Delta }V_{C}}$. Figure 3c shows the intrinsic potential difference ${\Delta V_{0}=V_{Si-3p}-V_{Ge-4p}}$ between the Si-3*p* and Ge-4*p* orbitals as a function of strain. ${\Delta V_0}$ linearly decreases with increasing lattice constant and becomes zero at the critical strain. The applied potential difference between the Si-3*s* and Ge-4*s* orbitals little affects the band gap. Thus, the strain-dependent on-site Coulomb potentials of the Si-3*p* and Ge-4*p* orbitals may be responsible for the quantum valley Hall states and the topological phase transition. Like an external electric field perpendicular to the layer, strain induces a potential difference between the A and B sublattices, leading to the topological phase transition.

The quantum valley Hall states described by a 2D Dirac Hamiltonian with mass gap is expected to have spontaneous electric polarization corresponding to the sublattice pseudospin polarization. Figure 4 shows the electric polarization and the Wannier function centers as a function of strain ${\Delta a}$ for ${E_z} =0$ (first column) and as a function of ${E_z}$ for $\Delta a=- \, 1.14\%$ (second column). In Fig. 4a,b, the band gap closure indicates the topological phase transition between $C_{V}=\pm \,1$. Figure 4c,d show the in-plane electric polarization ${P_a} \, (= {P_b})$ along the lattice vector ${\vec {a}} \, ({\vec {b}})$. We can see that the quantum valley Hall states with opposite valley Chern numbers have in-plane electric polarizations of opposite signs, and that the in-plane electric polarization undergoes abrupt inversion at the topological phase transition. ${|P_{a}|}$ depends very weakly on ${\Delta a}$ and ${E_Z}$, and is ${\sim 3.1\times 10^{-10}} \, {\hbox {C/m}}$, comparable to the values reported in monolayer Group-IV monochalcogenides.^22^ It is worth noting that ${|P_{a}|\simeq P_{0}/3=(2e)(a/3)}$, where ${P_0}$ is the quantum of electric polarization along ${\vec {a}}$. Considering that *a*/3 is the separation between the two sublattices, ${|P_{a}|}$ is equivalent to the electric dipole moment due to the charge carriers located on a sublattice, indicating that the in-plane electric polarization directly corresponds to the sublattice pseudospin polarization.

**Figure 4:**
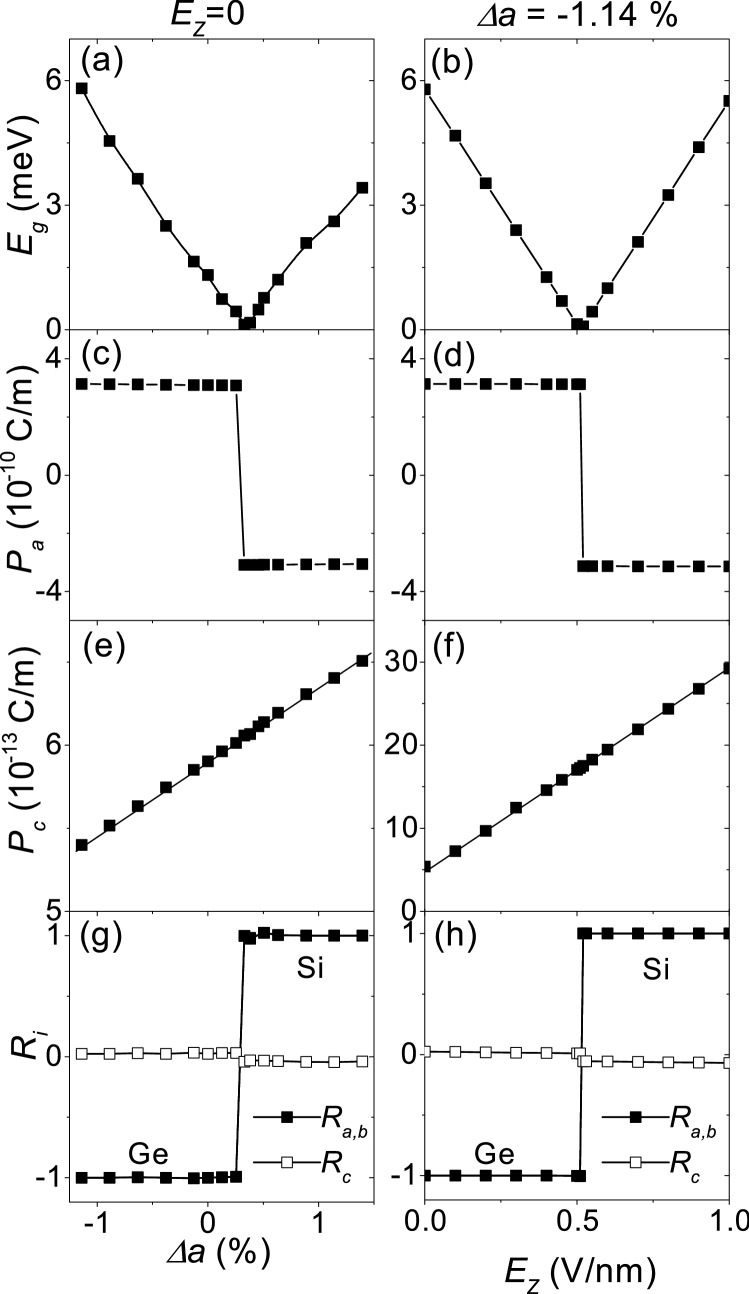
Electric polarization. The first and second columns correspond to $E_{Z}= 0$ and ${\Delta }a=- \, 1.14\%$, respectively. (**a**,**b**) Band gap ${E_g}$. (**c**,**d**) In-plane electric polarization ${P_a} \, (= {P_b})$. (**e**,**f**) Out-of-plane electric polarization ${P_c}$. (**g**,**h**) Wannier function center (${R_{a}}/3$, ${R_{b}}/3$, ${hR_c}$) in the fractional coordinates.

Figure 4e,f show the out-of-plane electric polarization ${P_c}$ along the lattice vector ${\vec {c}}$. ${P_c}$ is about ${0.6\times 10^{-12}} \, {\hbox {C/m}}$ at equilibrium, comparable to the values reported in previous works.^23, 24^ ${P_c}$ shows a linear response to ${\Delta a}$ and to ${E_z}$, and is independent of the topological phase transition, indicating that the out-of-plane electric polarization is independent of the sublattice pseudospin polarization.

Figure 4g,h show the sum of the maximally localized Wannier function centers of the occupied bands, ${\vec {R}_{WF}} = ({R_{a}}/3, {R_{b}}/3, {hR_c})$ in the fractional coordinates. When $R_{i}=\pm \,1$ ($i=a$, *b* or *c*), the Wannier function center has the same coordinate as that of the Si or Ge atom in the *i* direction. We can see that the Wannier function center is almost on the *ab*-plane with a very small out-of-plane component. The in-plane coordinates of the Wannier function center coincide with those of the Si or Ge atoms, undergoing an abrupt change at the topological phase transition. The Wannier function center, located only on the A or B sublattices (the Si or Ge atoms), corresponds to the sublattice pseudospin. Considering that the electronic contribution to the electric polarization is equal to ${2e\vec {R}_{WF}}$, we can confirm that the in-plane electric polarization is a purely electronic one and directly corresponds to the sublattice pseudospin polarization. The in-plane electric polarization can be expressed as ${P_{a}=(2e)(a/3)R_{a}}$, where $R_{a}=\pm \,1$ corresponding to the in-plane component of the Wannier function center serves as the sublattice pseudospin.

Because the number of gapless edge states is determined by the bulk topology according to the bulk-edge correspondence,^29, 30^ zigzag-edge nanoribbons were investigated for $\Delta a=-\, 1.14\%$ in order to further confirm the quantum valley Hall effect. The zigzag-edge nanoribbons are referred to as *N*-ZNR, where the ribbon width is represented by the number *N* of the Si-Ge pairs in the unit cell. The opposite edges of *N*-ZNR consist of Si and Ge atoms, respectively. Figure 5a shows the structure of H-terminated 8-ZNR and Fig. 5b shows the structure of 8-ZNR with H-terminated Ge edge and OH-terminated Si edge. Figure 5c shows the band structure of H-terminated 32-ZNR. Because of the tiny bulk gap, the band structure is similar to that of graphene nanoribbon.^31^

**Figure 5:**
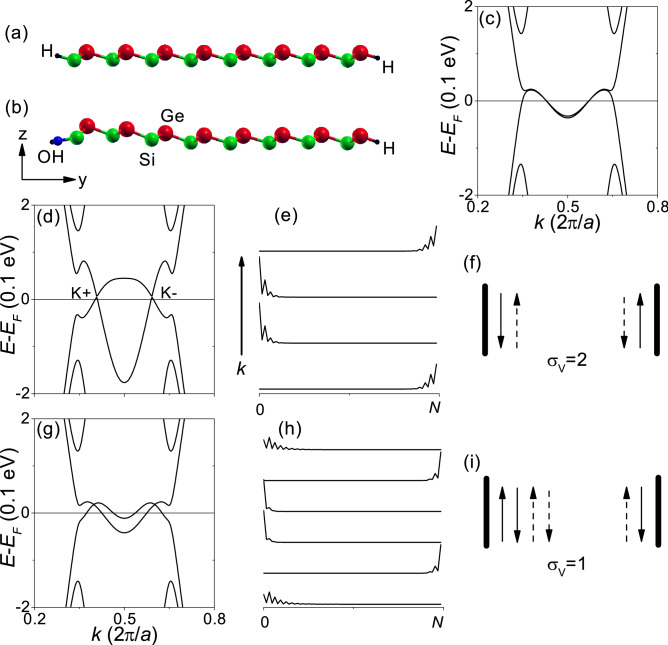
Zigzag-edge nanoribbons. Geometric structures of (**a**) H-terminated 8-ZNR and (**b**) 8-ZNR with H-terminated Ge edge and OH-terminated Si edge. Red, green, blue, and black balls correspond to Ge, Si, O, and H, respectively. (**c**) Band structure of H-terminated 32-ZNR. (**d**–**f**) H-terminated 32-ZNR with edge potential of ${-}$ 2 eV at the Si edge and + 2 eV at the Ge edge. (**g**–**i**) 32-ZNR with H-terminated Ge edge and OH-terminated Si edge. (**d**,**g**) Band structures. (**e**,**h**) The square of the wavefunction ${|\Psi |^2}$ at ${E_F}$. (**f**,**i**) Schematic for the propagating states at ${E_F}$. In (**f**,**i**), the up and down arrows correspond to the opposite propagating directions. The solid and dashed arrows represent the opposite valleys. ${\sigma _V}$ is a quantized valley Hall conductivity in units of ${e^{2}/h}$.

In a quantum valley Hall insulator, the bulk-edge correspondence is true only at the topological domain wall.^32^ Edge potentials at an edge can form a topological domain wall at the edge, where topologically protected gapless edge states are formed.^28, 33^ Nanoribbons inevitably have edge potentials, which can be induced by dangling ${\sigma }$-bonds and functional groups passivating the dangling bonds.^31, 33, 34^ Figure 5d–f correspond to H-terminated 32-ZNR with edge potential of -2 eV at the Si edge and +2 eV at the Ge edge (see Methods section), which forms a topological domain wall at each edge. The edge potential was chosen high enough that the topological confinement effect of the edge potential could exceed the finite size effect. As shown in Fig. 5d,e, there are gapless edge states within the bulk gap, which are well confined on an edge. Figure 5f shows a schematic for the propagating states at ${E_F}$. At each edge, backscattering of the gapless edge states is forbidden by the valley separation in the Brillouin zone because reversal of the propagating direction requires a reversal of the valley. At each edge, the number of gapless edge states per valley is 1 and is equal to the valley Chern number $C_{V}= 1$, which satisfies the bulk-edge correspondence.

Similar results can be obtained by using asymmetric edge terminations. Figure 5g–i correspond to 32-ZNR with H-terminated Ge edge and OH-terminated Si edge. As shown in Fig. 5g,h, there are gapless edge states within the bulk gap, which are well confined on an edge. Figure 5i shows a schematic for the propagating states at ${E_F}$. Only at the right edge (Ge edge), backscattering is forbidden and the number of gapless edge states per valley is equal to $C_{V}= 1$. By counting the number of the valley- and edge-resolved states propagating in a given direction, the quantized valley Hall conductivity ${\sigma _{V}}$ can be estimated in units of ${e^{2}/h}$. $\sigma _{V}= 2 \, {e^{2}/h}$ in Fig. 5f and $\sigma _{V}= 1 \, {e^{2}/h}$ in Fig. 5i.

To determine whether an in-plane electric field induces the topological phase transition between $C_{V}=\pm \,1$, we have investigated H-terminated 32 ZNR with an edge potential of -2 eV at the Si edge and +2 eV at the Ge edge under an in-plane transverse electric field ${E_y}$ as shown in Fig. 6. Figure 6a–c, d–f correspond to ${E_y} =0.5$ and -0.5 V/nm, respectively. Comparing Fig. 5d–f corresponding to ${E_y} =0$, we can see that the gapless edge states and the quantum valley Hall conductivity do not change under the electric fields, indicating that the in-plane transverse electric field does not induce a topological phase transition. As ${|E_{y}|}$ increases, the band crossing point near ${E_F}$ moves toward or away from ${k={\pi }/a}$ as shown in Fig. 6a,d. As ${|E_{y}|}$ further increases, the band gap opens as shown in Fig. 6g,h corresponding to ${|E_{y}|} =1.5 \, {\hbox {V/nm}}$.

**Figure 6:**
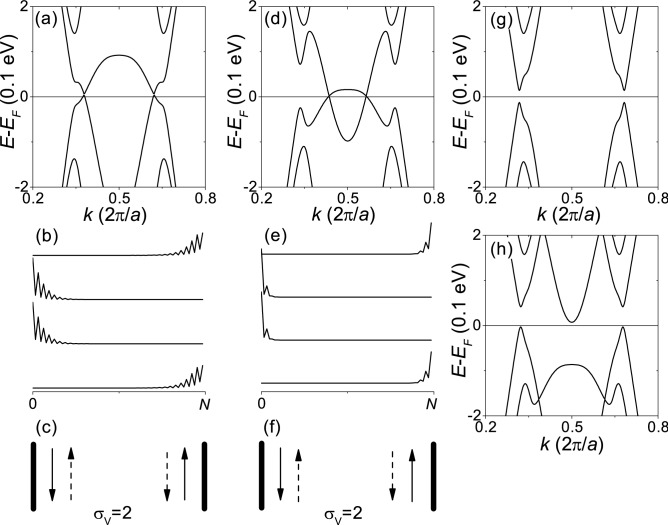
Zigzag-edge nanoribbons under a transverse electric field. H-terminated 32-ZNR with edge potential of ${-}$ 2 eV at the Si edge and + 2 eV at the Ge edge. (**a**–**c**) ${E_y} =0.5 \, {\hbox {V/nm}}$. (**d**–**f**) ${E_y} =-\,0.5 \, {\hbox {V/nm}}$. (**g**) ${E_y} =1.5 \, {\hbox {V/nm}}$. (**h**) ${E_y} =-\,1.5 \, {\hbox {V/nm}}$. (**a**,**d**,**g**,**h**) Band structures. (**b**,**e**) ${|\Psi |^2}$ at ${E_F}$. (**c**,**f**) Schematics for the propagating states at ${E_F}$. In (**c**,**f**), the up and down arrows correspond to the opposite propagating directions. The solid and dashed arrows represent the opposite valleys. ${\sigma _V}$ is a quantized valley Hall conductivity in units of ${e^{2}/h}$.

To summarize, we have investigated tiny-gap semiconductor SiGe monolayer by using the density functional theory calculations. We found that the SiGe monolayer is a quantum valley Hall insulator with spontaneous electric polarization, undergoing a topological phase transition under a small biaxial strain. Strain-dependent on-site Coulomb potentials of the Si-3*p* and Ge-4*p* orbitals seem to be responsible for the quantum valley Hall state and the topological phase transition. The topological phase transition entails abrupt inversion of the in-plane electric polarization directly corresponding to inversion of the sublattice pseudospin polarization, while the out-of-plane electric polarization shows a linear response regardless of the topological phase transition. Thus, the quantum valley Hall state described by a two-dimensional Dirac Hamiltonian with mass gap entails in-plane ferroelectricity corresponding to a sublattice pseudospin ferromagnetism.

## Methods

A SIESTA package,^35^ which uses a localized linear combination of the numerical atomic-orbital basis sets, was employed for the DFT calculations. A generalized gradient approximation of Perdew-Burke-Ernzerhof was used for the exchange and correlation potential.^36^ A double-${\zeta }$ polarized basis set and norm-conserving fully-relativistic pseudopotentials were used. The plane-wave cutoff energy of 350 Ry and *k*-points of $100 \times 100\times 1$ meshes in a Monkhorst-Pack scheme were used. The atomic coordinates were optimized by using the conjugated gradients method with a maximum force tolerance of 0.1 eV/nm. The vacuum spacing for the SiGe monolayer was set to 2.6 nm. Zigzag-edge nanoribbons were considered as a one-dimensional system periodic in a zigzag direction. A periodic sawtooth potential was used to simulate the external electric fields ${E_y}$ and ${E_z}$ applied along the *y* and *z* axes, respectively. If not specified, the spin–orbit coupling is neglected.

To apply a staggered AB-sublattice potential, the LDA+U method implemented in SIESTA was used with the option that the *U* parameter is interpreted as a local potential shift.^37^ The applied potential difference between the Ge-4*p* and Si-3*p* orbitals corresponds to ${{\Delta }V} = {U_{Ge-4p}}{\hbox {-}} {U_{Si-3p}}$, where ${U_{Ge-4p}}$ and ${U_{Si-3p}}$ are the *U* parameters for the Ge-4*p* and Si-3*p* orbitals, respectively. In zigzag-edge nanoribbons, edge potentials were applied by using ${U_{Ge-4p}}$ and ${U_{Si-3p}}$ at the edge sites. The macroscopic electric polarization was calculated by using the Berry phase approach implemented in SIESTA.^38^ The electric polarization is only defined modulo a quantum of the polarization ${P_{0}=2eR}$, where *e* and *R* are the elementary charge and an arbitrary lattice vector, respectively. We chose the values between ${{\pm }\,P_{0}/2}$ as the electric polarization. The electric polarization was normalized by the cell area and is given in units of C/m.

Using the wavefunctions obtained from the DFT calculations, maximally localized Wannier functions were constructed within the Wannier90 code.^39^ Berry curvatures were calculated based on the Wannier interpolation. Using the anomalous Hall conductivity calculation routine implemented in Wannier90 code, the valley-resolved Chern number ${C(\tau )}$ was calculated by integrating the Berry curvature near a valley. The valley Chern number was obtained as ${C_{v}=C({\mathrm{K+}})-C({\mathrm{K-}})}$. ${K+}$ and ${K-}$ represent the inequivalent K points in the Brillouin zone, corresponding to the opposite valleys.

## References

[1] Novoselov KS, Geim AK, Morozov SV, Jiang D, Zhang Y, Dubonos SV, Grigorieva IV, Firsov AA (2004). Electric field effect in atomically thin carbon films. Science.

[2] Novoselov KS, Geim AK, Morozov SV, Jiang D, Katsnelson MI, Grigorieva IV, Dubonos SV, Firsov AA (2005). Two-dimensional gas of massless Dirac fermions in graphene. Nature.

[3] Kane CL, Mele EJ (2005). Quantum spin Hall effect in graphene. Phys. Rev. Lett..

[4] Molle A, Goldberger J, Houssa M, Xu Y, Zhang S-C, Akinwande D (2017). Buckled two-dimensional Xene sheets. Nat. Mater..

[5] Şahin H, Cahangirov S, Topsakal M, Bekaroglu E, Akturk E, Senger RT, Ciraci S (2009). Monolayer honeycomb structures of group-IV elements and III–V binary compounds: first-principles calculations. Phys. Rev. B.

[6] Zhang Z, Liu X, Yu J, Hang Y, Li Y, Guo Y, Xu Y, Sun X, Zhou J, Guo W (2016). Tunable electronic and magnetic properties of two-dimensional materials and their one-dimensional derivatives. Comput. Mol. Sci..

[7] Liu C, Feng W, Yao Y (2011). Quantum spin Hall effect in silicene and two-dimensional germanium. Phys. Rev. Lett..

[8] Yu X, Wu J (2018). Evolution of the topological properties of two-dimensional group IVA materials and device design. Phys. Chem. Chem. Phys..

[9] Ezawa M (2015). Monolayer topological insulators: Silicene, germanene, and stanene. J. Phys. Soc. Jpn..

[10] Lü T-Y, Liao X-X, Wang H-Q, Zheng J-C (2012). Tuning the indirect-direct band gap transition of SiC, GeC and SnC monolayer in a graphene-like honeycomb structure by strain engineering: A quasiparticle GW study. J. Mater. Chem..

[11] Wickramaratne D, Weston L, Van de Walle CG (2018). Monolayer to bulk properties of hexagonal boron nitride. J. Phys. Chem. C.

[12] Zhou H, Zhao M, Zhang X, Dong W, Wang X, Bu H, Wang A (2013). First-principles prediction of a new Dirac-fermion material: Silicon germanide monolayer. J. Phys.: Condens. Matter.

[13] Padilha JE, Seixas L, Pontes RB, da Silva AJR, Fazzio A (2013). Quantum spin Hall effect in a disordered hexagonal $${\text{Si}}_x {\text{ Ge }}_{1-x}$$ alloy. Phys. Rev. B.

[14] Choi S-M, Jhi S-H, Son Y-W (2010). Effects of strain on electronic properties of graphene. Phys. Rev. B.

[15] Yan J-A, Gao S-P, Stein R, Coard G (2015). Tuning the electronic structure of silicene and germanene by biaxial strain and electric field. Phys. Rev. B.

[16] Qin R, Zhu W, Zhang Y, Deng X (2014). Uniaxial strain-induced mechanical and electronic property modulation of silicene. Nanoscale Res. Lett..

[17] Wang Y, Ding Y (2013). Strain-induced self-doping in silicene and germanene from first-principles. Solid State Commun..

[18] Behzad S (2018). Effect of uni-axial and bi-axial strains and vertical electric field on free standing buckled germanene. J. Electron. Spectrosc..

[19] Zhao H (2012). Strain and chirality effects on the mechanical and electronic properties of silicene and silicane under uniaxial tension. Phys. Lett. A.

[20] Sakib, H., Ahmed, T. & Subrina, S. Uniaxial strain on monolayer SiGe: Strain tunable electronic properties. *10th International Conference on Electrical and Computer Engineering* 313-316 (IEEE, 2019). 10.1109/ICECE.2018.8636776.

[21] Mikhailov SA (2013). Ferroelectric instability of two-dimensional crystals. Phys. Rev. B.

[22] Fei R, Kang W, Yang L (2016). Ferroelectricity and phase transitions inmonolayer group-IV monochalcogenides. Phys. Rev. Lett..

[23] Ma X, Ai H, Gao H, Zhang X, Lia W, Zhao M (2019). Valley polarization and ferroelectricity in a two-dimensional $${\text{ GaAsC }}_6$$ monolayer. Phys. Chem. Chem. Phys..

[24] Sante DD, Stroppa A, Barone P, Whangbo M-H, Picozzi S (2015). Emergence of ferroelectricity and spin-valley properties in two-dimensional honeycomb binary compounds. Phys. Rev. B.

[25] Georgi A (2017). Tuning the pseudospin polarization of graphene by a pseudomagnetic field. Nano Lett..

[26] Rozhkova AV, Sboychakova AO, Rakhmanova AL, Nori F (2016). Electronic properties of graphene-based bilayer systems. Phys. Rep..

[27] Xiao D, Yao W, Niu Q (2007). Valley-contrasting physics in graphene: Magnetic moment and topological transport. Phys. Rev. Lett..

[28] Yao W, Yang SA, Niu Q (2009). Edge states in graphene: From gapped flat-band to gapless chiral modes. Phys. Rev. Lett..

[29] Hatsugai Y (1993). Chern number and edge states in the integer quantum Hall effect. Phys. Rev. Lett..

[30] Qi X-L, Wu Y-S, Zhang S-C (2006). General theorem relating the bulk topological number to edge states in two-dimensional insulators. Phys. Rev. B.

[31] Lee KW, Lee CE (2018). Transverse electric field-induced quantum valley Hall effects in zigzag-edge graphene nanoribbons. Phys. Lett. A.

[32] Li J, Morpurgo AF, Büttiker M, Martin I (2010). Marginality of bulk-edge correspondence for single-valley Hamiltonians. Phys. Rev. B.

[33] Lee KW, Lee CE (2017). Topological confinement effect of edge potentials in zigzag-edge graphene nanoribbons under a staggered bulk potential. Curr. Appl. Phys..

[34] Yamanaka A, Okada S (2017). Polarity control of h-BN nanoribbon edges by strain and edge termination. Phys. Chem. Chem. Phys..

[35] Sánchez-Portal D, Ordejon P, Artacho E, Soler JM (1997). Density-functional method for very large systems with LCAO basis sets. Int. J. Quantum Chem..

[36] Perdew JP, Burke K, Ernzerhof M (1996). Generalized gradient approximation made simple. Phys. Rev. Lett..

[37] Dudarev SL, Botton GA, Savrasov SY, Humphreys CJ, Sutton AP (1998). Electron-energy-loss spectra and the structural stability of nickel oxide: An LSDA+U study. Phys. Rev. B.

[38] King-Smith RD, Vanderbilt D (1993). Theory of polarization of crystalline solids. Phys. Rev. B.

[39] Mostofi AA, Yates JR, Lee Y-S, Souza I, Vanderbilt D, Marzari N (2008). Wannier90: A tool for obtaining maximally-localised Wannier functions. Comput. Phys. Commun..

